# Ultrafiltration biofeedback guided by blood volume monitoring to reduce intradialytic hypotensive episodes in hemodialysis: study protocol for a randomized controlled trial

**DOI:** 10.1186/1745-6215-15-483

**Published:** 2014-12-10

**Authors:** Kelvin CW Leung, Robert R Quinn, Pietro Ravani, Jennifer M MacRae

**Affiliations:** Department of Medicine, University of Calgary, Calgary, Canada; Department of Community Health Sciences, University of Calgary, Calgary, Canada

**Keywords:** Hemodialysis, Blood volume monitoring biofeedback, Intradialytic hypotension, Randomized crossover trial

## Abstract

**Background:**

Fluid removal during dialysis, also known as ultrafiltration (UF), leads to intradialytic hypotension (IDH) in a significant number of patients treated with hemodialysis (HD) and is associated with an increase in morbidity and mortality. At present, there are no accepted standards of practice for the prevention or treatment of IDH. Relative blood volume monitoring (BVM) is based on the concept that the hematocrit increases with UF, relative to the patient’s baseline hematocrit. The use of BVM biofeedback, whereby the HD machine automatically adjusts the rate of UF based on the relative blood volume, has been proposed for the prevention of IDH.

**Methods/Design:**

This is a 22-week randomized crossover trial. Participants undergo a 4-week run-in phase to standardize medications and dialysis prescriptions. Subsequently, participants are randomized to standard HD or to BVM biofeedback for a period of 8 weeks followed by a 2-week washout phase before crossing over. The dialysis prescription remains identical for both arms. The primary outcome is the frequency of symptomatic IDH as defined by an abrupt drop in the systolic blood pressure of ≥20 mm Hg accompanied by headache, dizziness, loss of consciousness, thirst, dyspnea, angina, muscle cramps or vomiting. Secondary outcomes include the number of symptomatic IDH episodes and any reduction in IDH episodes, nursing interventions, dialysis adequacy, total body water, extra- and intracellular fluid volumes, brain natriuretic peptide and cardiac troponin levels, blood pressure, antihypertensive medication use, patient symptoms and quality of life.

**Discussion:**

Our study will determine the impact of using BVM biofeedback to prevent IDH and other serious adverse events in susceptible patients.

**Trial registration:**

Clinicaltrials.gov NCT01988181 (6 November 2013).

**Electronic supplementary material:**

The online version of this article (doi:10.1186/1745-6215-15-483) contains supplementary material, which is available to authorized users.

## Background

The majority of patients with end-stage renal disease are treated with hemodialysis (HD) to regulate their fluid balance and other native kidney functions [1, 2]. Patients treated with HD have a 5-year mortality rate of 57%, with the majority of deaths due to cardiac and infectious causes [3, 4]. A growing body of literature has emerged linking chronic fluid overload in HD to hypertension, left ventricular hypertrophy and increased all-cause mortality [5–7]. To prevent volume overload and maintain fluid balance, 1 to 5 L of fluid must be removed during each HD treatment.

Rapid fluid removal, or ultrafiltration (UF), during a short period of time can lead to intradialytic hypotension (IDH) in as many as 25% to 50% of patients treated with HD [8]. IDH is most commonly defined as an abrupt drop in systolic blood pressure of ≥20 mm Hg accompanied by symptoms of cerebral, cardiac, gastrointestinal or musculoskeletal ischemia [9–11]. Observational studies have shown that IDH or a drop in post-dialysis systolic blood pressure is associated with an increase in morbidity and mortality [12–20].

UF during HD leads to a fall in the patient’s blood volume. The blood volume can be measured by tracking the changes in hemoglobin or protein concentration at the arterial port during dialysis using optical photometry or ultrasound [21–28]. In clinical studies, higher rates of UF lead to faster declines in the relative blood volume and as a result, a steeper decline in the blood volume monitoring (BVM) curves [27–29]. BVM-guided biofeedback is based on the principle that the relative blood volume (compared to baseline) appears to decrease more rapidly in sessions complicated by IDH [25, 30]. As a result, BVM-guided UF biofeedback, whereby the dialysis machine automatically reduces the rate of UF prior to reaching the patient’s critical blood volume, has been proposed for the prevention of IDH and its sequelae [22, 23, 31–34].

We will conduct a randomized controlled clinical trial to test whether biofeedback incorporating BVM-guided UF adjustments alone (without adjustment of the dialysate sodium concentration), in addition to best clinical practice, results in a reduction in the frequency of symptomatic IDH episodes and patient symptoms compared to best clinical practice alone. Secondary outcomes include the frequency of IDH-related interventions, dialysis-related symptoms, dialysis adequacy, volume control, biomarkers of volume overload and cardiac stress, blood pressure and quality of life.

## Methods/Design

### Study design

**Figure 1:**
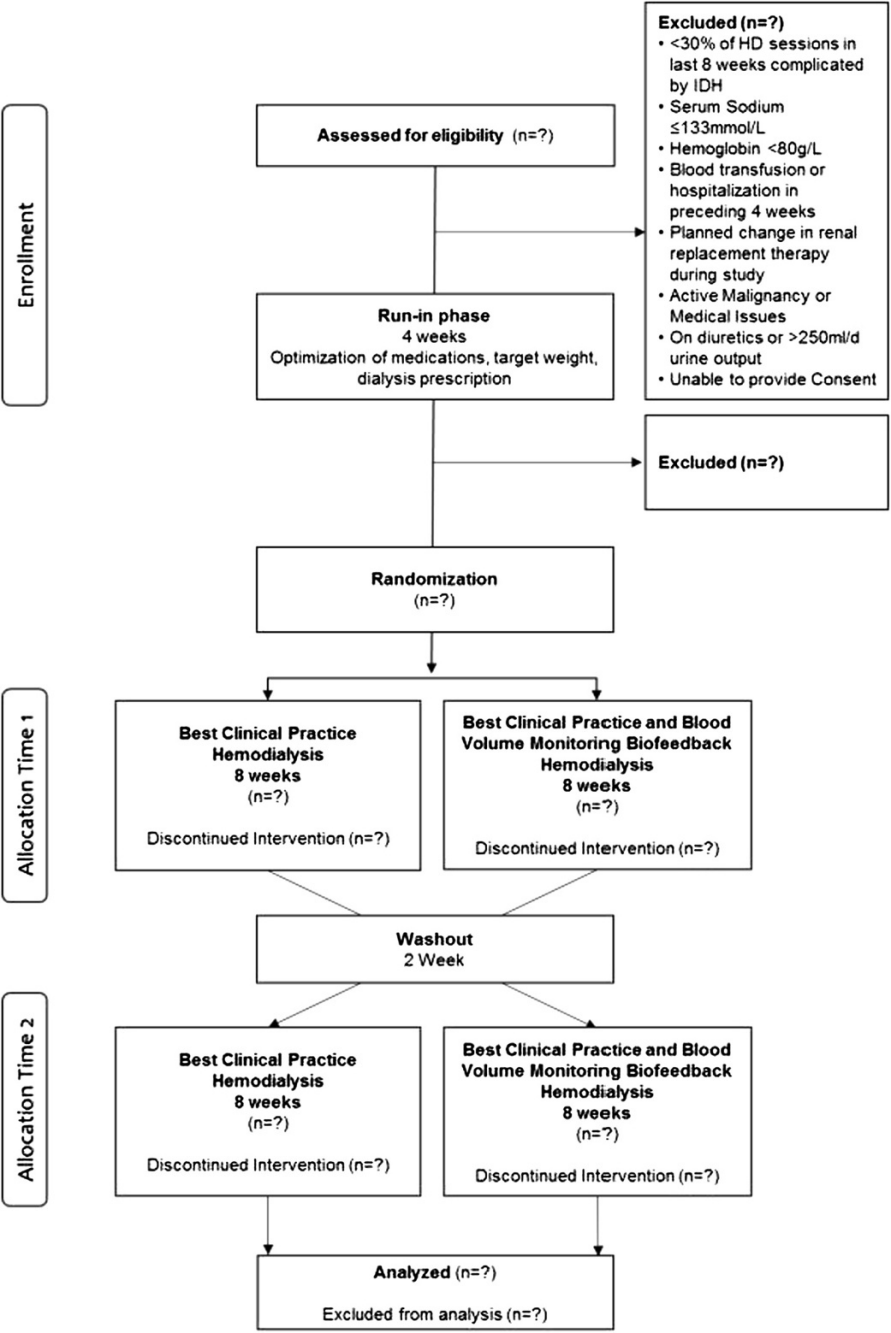
**Participant flow diagram.** HD, hemodialysis; IDH, intradialytic hypotension.

This is a 22-week parallel-group randomized crossover trial. During the first part of the study, the run-in and dialysis optimization phase, eligible participants will undergo a 4-week run-in. During this period, all participants will undergo a comprehensive clinical assessment including a clinical weight assessment, an antihypertensive medication review and standardization of their dialysis prescription. At the end of the run-in phase, participants that still meet the eligibility criteria will enter part 2, the randomized crossover phase. In this phase, the participants are randomized to regular best clinical practice HD (without BVM-guided UF biofeedback; the control arm) or to best clinical practice plus BVM-guided UF biofeedback (the intervention arm) for an 8-week period. This will be followed by a 2-week washout phase and then the participants will be crossed over for a second 8-week phase. The participant flow chart and timeline are shown in Figure 1. The study will be conducted and reported following the Consolidated Standards of Reporting Trials (CONSORT) 2010 guidelines [35]. The study protocol was approved by the University of Calgary Conjoint Health Research Ethics Board (ethics ID: REB13-1139).

### Study setting

The clinical trial will be held at two tertiary care units (Foothills Medical Centre and Peter Lougheed Centre) and three community HD units (Fanning Centre, Sheldon Chumir Centre and Sunridge Centre) in the Southern Alberta Renal Program, Calgary, Alberta, Canada.

### Study participants

All participants who have been treated with HD for more than 3 months will be screened for eligibility. To be eligible for the study, participants must be >18 years of age, medically stable, undergo HD three to four times per week for a minimum of 3 hours per session, and have had >30% of their HD sessions in the preceding 8 weeks complicated by symptomatic IDH. Participants with serum sodium ≤133 mmol/L [36], hemoglobin <80 g/L, active malignancy, a history of blood transfusion or hospitalization in the preceding 4 weeks, routine use of diuretics for volume management, a history of ongoing urine output estimated at greater than or equal to 250 mL (one cup) per day, or a planned change in the renal replacement modality during the study period will be excluded. Informed consent will be obtained from each individual who agrees to participate in the study.

### Definition of symptomatic intradialytic hypotension

Symptomatic IDH is defined as a drop in systolic blood pressure of ≥20 mm Hg from baseline with associated symptoms [9, 10]. Symptoms include sudden-onset headache, dizziness, unconsciousness, thirst, dyspnea, angina, muscle cramps and vomiting [10, 11].

### Interventions

#### Part 1: run-in phase

Following enrollment, participants will undergo a 4-week run-in phase to optimize their dialysis weight and dialysis prescriptions, and to determine the critical relative blood volume values (BVM will be enabled). During the first 2 weeks of the run-in phase, participants will undergo a medication review and dry-weight reduction based on a modified protocol from the Dry-Weight Reduction In Hypertensive Hemodialysis Patient (DRIP) trial [37, 38].

#### Part 2: crossover study phase

Participants that continue to have more than 30% of their sessions complicated by symptomatic IDH during the run-in phase are randomized to either 8 weeks of best clinical practice (without BVM or biofeedback; control) or best clinical practice plus BVM-guided UF biofeedback (intervention), followed by a 2-week washout period (using the control phase HD prescription) before crossing over to the other study arm for a second 8-week block. Participants will have clinical assessments of their dry weight at the beginning of each week.

#### Control group

All study participants will be dialyzed with the Fresenius 5008 HD machine (Fresenius Medical Care, Bad Homburg, Germany) using high-flux dialyzers. Participants in the best clinical practice (control) group will use the same prescription used during the run-in phase: dialysate sodium of 138 mmol/L, dialysate calcium of 1.25 mmol/L, dialysate temperature of 36°C and constant UF rate. BVM will be disabled in this group.

#### Intervention group

Participants in the BVM-guided UF biofeedback group will have the same prescription as the control group, but will also have the UF rate automatically adjusted by the Fresenius 5008 HD machine based on changes in relative blood volume.

### Ultrafiltration biofeedback guided by blood volume monitoring using the Fresenius 5008

**Figure 2:**
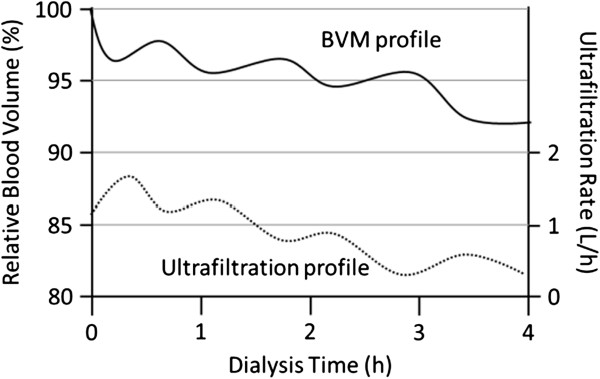
**Depiction of blood volume monitoring (BVM) profile and the corresponding ultrafiltration profile.**

The Fresenius 5008 uses an ultrasound monitor incorporated into the machine to detect ultrasonic velocity changes to derive the total protein concentration, which is a sum of total plasma proteins and hemoglobin. A temperature monitor is also incorporated to correct for temperature-related changes in ultrasound velocity [21, 39]. Since the total protein does not change, any changes in its concentration are attributed to blood volume changes (Figure 2). The relative blood volume is calculated by dividing the initial concentration of total protein by the total protein concentration at any given time, multiplied by 100 [21]. This method for measuring relative blood volume has been previously validated with both optical and laboratory hemoglobin techniques [21, 24].

The HD software in the Fresenius 5008 HD machine adjusts the UF rate based on the critical blood volume entered at the beginning of the dialysis session for each individual patient. There is no adjustment to the dialysate sodium concentration. To determine the actual UF rate, the HD software first calculates the maximum UF rate. The maximum UF rate is 2 times the total UF divided by the remaining time. The actual UF rate is the maximum UF rate multiplied by the UF coefficient, which is a number between zero and one. To allow for the maximum UF at the onset of HD, the UF coefficient is one at the start of HD and continues to remain at one until the relative blood volume is halfway towards the critical relative blood volume. At the halfway point between the start and the critical relative blood volume, the UF coefficient decreases linearly. Once the critical relative blood volume is reached, the UF coefficient becomes zero, resulting in cessation of UF [21, 24, 40].

### Determination of critical blood volume

There is currently no standardized method for determining the critical relative blood volume. To standardize this process, a single investigator (KL) will use the following method/algorithm:

1. Identify the most recent episode of symptomatic IDH as per the study definition.
2. The critical blood volume will be equal to the relative blood volume recorded immediately prior to the episode of symptomatic IDH.
3. The critical blood volume will be reassessed weekly.

### Weight adjustments

Each week participants will have their target weight assessed by a single study investigator (KL). The rounding dialysis physician will be encouraged to discuss any weight adjustments with study personnel.

### Responding to episodes of intradialytic hypotension

In the event that an IDH episode occurs, the bedside dialysis nurse will follow a predefined IDH algorithm (Additional file 1) [38]. Following resolution of an IDH episode as defined in the primary outcome section, UF will resume. In the control group, a constant UF rate will be reset to meet the UF goal. In the intervention group, BVM-guided UF biofeedback will be re-enabled.

### Outcomes

#### Primary outcome

The primary outcome is the rate of symptomatic IDH. The number of symptomatic IDH episodes along with the duration of each dialysis treatment will be captured. The rate of IDH for each session will be calculated by dividing the number of episodes by the duration of the session in hours. The rate of IDH will be calculated for every dialysis treatment. The rate of symptomatic IDH will be measured in the 2 months preceding enrolment and during each phase of the study. By using the rate of symptomatic IDH, rather than the frequency or number of IDH episodes per session as done in previous studies, we will be more sensitive to meaningful changes in IDH episodes as multiple episodes of symptomatic IDH can occur over various durations of dialysis [31, 32, 41].

#### Secondary outcomes

The secondary outcomes of interest are the number and frequency of nursing interventions, the number and frequency of both symptomatic and asymptomatic IDH, the number and frequency of dialysis symptoms, dialysis adequacy as measured by single-session *Kt*/*V*, total body water, extracellular fluid volume (ECFV), intracellular fluid volume (ICFV), the ECFV:ICFV ratio as determined by electrical bioimpedance, changes in brain natriuretic peptide (BNP) level, changes in high-sensitivity-cardiac troponin level, changes in blood pressure (mean arterial pressure pre-, intra- and post-dialysis), changes in antihypertensive medications, changes in the BVM curves, intradialytic and interdialytic symptom survey, and nursing satisfaction.

IDH-related nursing interventions are defined as the use of the Trendelenburg/supine position, discontinuation of UF, fluid/saline challenge, return of blood, shortened dialysis run, or a prolonged (more than 15 minutes) post-dialysis recovery period as a result of IDH or its symptoms. Given the subjective nature of IDH-related symptoms and intervention, in-services and customized dialysis run sheets will be provided to all nurses at participating dialysis centers to ensure that all events are recorded, and recorded consistently.

Dialysis adequacy, measured by single-pool *Kt*/*V*, will be measured and recorded (usual care) at the end of each dialysis session on the session sheet. Single-pool *Kt*/*V* will be calculated using the previously validated, online clearance measurement method, which detects changes in conductivity in the dialysate to reflect the clearance of serum electrolytes and urea [42].

Whole body and segmental bioimpedance analysis has been validated for the determination of fluid composition in HD patients [43–47]. Electrical bioimpedance will be performed during the mid-week HD session of weeks 1, 4, 8, 12, 14, 18 and 22 of the study to determine the total body water, ECFV and ICFV levels and the ECFV:ICFV ratio.

Biomarkers of cardiac stress, serum BNP and high-sensitivity cardiac troponin levels as well as the number and class of antihypertensive medications used will be recorded at the mid-week HD session of weeks 1, 4, 12, 14 and 22 of the study. Diuretic use will not be recorded as participants with significant residual renal function are excluded.

Two types of survey will be administered and completed by the patient alone, or with the aid of the dialysis nurse. The first, provided at the beginning of each dialysis session, is a validated survey inquiring about the time it took the patient to recover to baseline following a previous dialysis session (interdialytic period) [48]. The second, provided at the end of each dialysis session, inquires about intradialytic symptoms of IDH, specifically nausea, vomiting, chest pain, shortness of breath, headache, muscle cramps, dizziness, fainting, fatigue and anxiety. A survey inquiring about nursing perception and attitudes of the BVM-guided UF biofeedback will be provided to the nurses at the end of the control and intervention phases.

### Sample size calculation

We have previously estimated that 23% of our HD population have IDH [38]. To estimate the sample size, we conservatively assumed that only one IDH episode would occur per run (although it is likely that more than one episode will occur per run, increasing the study power). With this approach, and using a 2 × 2 crossover design, we estimated that a sample size of 30 participants would provide a power of 90% to demonstrate a 30% reduction in the rate of IDH under the biofeedback treatment vs. the control treatment, with a two-sided alpha of 0.05. We will enroll 34 participants to guard against dropout, which can be as high as 30% [41]. These estimates are based on simulation studies assuming only one IDH episode per run and two Poisson processes with average rates of IDH equal to 7 (control) and 5 (intervention) episodes over 24 runs. We used R (R Foundation for Statistical Computing, Vienna, Austria) [49] for simulations, and the package clusterPower in R.

### Recruitment

Recruitment will take place sequentially at the four participating HD units. From pilot data, we anticipate that approximately 20% of the 400 HD patients in participating units will meet the criteria for IDH [38] and at least half of the eligible participants (40 participants) will be willing to participate in our study [50, 51].

### Assignment of intervention, allocation concealment mechanism and implementation

Randomization of allocation sequence will be done using computer-generated random numbers under the supervision of a statistician in the Department of Medicine, University of Calgary. Subsequently, the allocation sequence will be inserted into sequentially numbered, opaque, sealed envelopes. Following study consent and enrollment, an envelope containing the allocation sequence will be provided to the patient’s HD nurse, who will implement the intervention according to the protocol.

### Blinding

Given the nature of our intervention, and the inability to disable the BVM-guided UF biofeedback screens, prompts and alarms, we will not be able to blind the study personnel (KL) or the bedside dialysis nurse. The trial participants will be blinded to the intervention. To reduce bias, the HD nurse will be encouraged to document all intradialytic symptoms and interventions, as well as not to reveal the allocation sequence to the trial participant. In addition, study personnel (KL) will assess target weights and administer surveys uniformly. Study personnel involved with data abstraction and analysis will be blinded to the study intervention.

### Data collection

#### Collection of baseline and run-in phase data

Baseline patient demographics (age, gender, HD vintage and race), comorbidities (e.g. congestive heart failure, diabetes mellitus and peripheral vascular disease), cause of renal disease, medications (number and class of antihypertensive), laboratory investigations (e.g. serum electrolytes, complete blood count and albumin), HD prescription (dialyzer type, composition, temperature, blood and dialysate flow rates, target weight, anticoagulation, use of sodium and/or UF profiles), and number of episodes of IDH over the preceding 8 weeks and run-in phase will be extracted from HD charts and local electronic health records by the study investigator.

#### Collection of primary outcome variables

Both sitting and standing blood pressure will be measured in a standardized fashion at the beginning and end of each dialysis session [52, 53]. Intradialytic blood pressures will be measured in a sitting position every 30 minutes and at the time of IDH-related symptoms as defined in the outcomes section. Sitting blood pressures will be measured with the patient seated with their feet flat on the floor or a foot rest, back against a chair with a bare arm resting on a support, whereby the midpoint of the upper arm is at the level of the heart. Standing blood pressures will be measured with the patient standing feet flat on the floor, with a bare arm resting on a support, whereby the midpoint of the upper arm is at the level of the heart. An appropriate-sized blood pressure cuff, where the cuff bladder length is 80% of the arm circumference, will be used. Blood pressure taken while the patient is in a Trendelenburg or supine position will be accepted if the patient is unable to sit during the pre-specified blood pressure measurement or is experiencing IDH-related symptoms. All blood pressures will be measured using an automated cuff attached to the dialysis machine. Manual blood pressure will be accepted if an automated blood pressure measurement cannot be obtained. Blood pressure, method of measurement, patient position, IDH-related symptoms and nursing interventions will be recorded by the bedside dialysis nurse.

#### Collection of secondary outcome variables

Dialysis adequacy, bioimpedance, BNP, high-sensitivity cardiac troponin, intra- and interdialytic symptom survey and antihypertensive medication use will be collected by study personnel at the pre-specified times (Table 1).

**Table 1:** **Data collection schedule**

		Part 1			Part 2			Part 2	
	Every session	Mid-week 1	Mid-week 4		Mid-week 8	Mid-week 12		Mid-week 18	Mid-week 22
Hemodialysis run sheet	X			Randomization			Crossover and washout		
*Kt*/*V*	X								
Electrical bioimpedance		X	X		X	X		X	X
Brain natriuretic peptide		X	X		X	X		X	X
High-sensitivity cardiac troponin		X	X		X	X		X	X
Intradialytic symptom survey	X								
Interdialytic symptom survey	X								
Medication review		X	X		X	X		X	X

### Participant retention and follow-up

Following enrollment, every effort will be made to follow up participants until the end of the study phase. Participants moving to different dialysis sites across the city of Calgary will be followed unless they move to a site not using the Fresenius 5008 machines, at which point they will be censored. Participants who move out of the city will be censored.

### Statistical analysis

We will use mixed-effects Poisson regression to test the null hypothesis that the ratio of the rate of IDH episodes (main model exposure) during the intervention phase over the rate of IDH episodes during the control phase will range between 0.7 and 1.3 (i.e., the alternative two-sided hypothesis is that the incidence rate ratio will be 0.7 or lower). We will study the effect of the intervention as a fixed effect and account for the correlation in the data due to the subject using random effects. The multi-level model will have errors due to within-subject variation and due to between-cluster variation (between subject variance). In the event that the study generates over-dispersed data, we will use a negative binomial regression with the approach as described above. We will also explore whether participants with larger drops in systolic blood pressure (>30 mmHg) benefit from the planned intervention. They will be analyzed as a subgroup.

### Ethics

Ethical approval has been granted from the University of Calgary Research Ethics Board. The research coordinator will determine patient eligibility, obtain consent from participants for participation in the study and access the sealed envelope containing allocation details. The coordinator will obtain the HD run sheets and submit them to an independent data entry clerk who is blinded to allocation. All data are kept strictly confidential. The principal investigator (JM) and study personnel (KL) are responsible for coordination of the study. The trial is registered with Clinicaltrials.gov (NCT01988181).

## Discussion

Randomized studies assessing the role of BVM and BVM-guided UF biofeedback in the prevention of IDH are limited and of low quality. One the largest studies assessing BVM *without* biofeedback, which was done by Reddan *et al.*, randomized 443 participants to BVM or conventional HD over a 6-month period. Participants in the BVM group had UF rates adjusted at the discretion of the bedside dialysis nurse with a suggested UF algorithm. Due to the need for manual adjustment of the UF rate, it is unclear if the nurses promptly responded to changes in relative blood volume using the suggested algorithm. In fact, the participants randomized to BVM had increased hospitalization and mortality, which may have reflected a lack of adherence to the UF algorithm, or alternatively could have been related to more cautious fluid removal due to the feedback provided by the BVM device [33].

However, when BVM is combined with biofeedback there may be a beneficial reduction in IDH and blood pressure [31, 32, 41, 54]. In a 16-week randomized crossover study by Dasselaar *et al.*, 28 hypertensive patients were randomized to either BVM-guided UF and sodium biofeedback or standard HD for blood pressure and volume control. Despite a significant reduction in blood pressure of 22.5/8.3 mm Hg in the BVM group and extracellular water to body volume ratios when compared to the standard HD group, the overall weight did not change. Although the values were not reported, this study also found a statistically significant reduction in dialysis hypotension [54]. A recent meta-analysis of six studies using BVM-guided biofeedback reported a 39% overall reduction in the number of dialysis sessions complicated by IDH [55]. Of the six studies assessing IDH, three did not have IDH as the primary outcome. Five studies employed an intervention that included both dialysate sodium and UF biofeedback [31, 34, 56–58]. The combination of dialysate sodium and UF biofeedback over UF biofeedback alone raises concerns regarding the potential for sodium loading and resultant interdialytic weight gain through increased plasma osmolality and thirst, perpetuating the vicious cycle of IDH. In addition, the duration of the intervention phase in these studies ranged from 2 weeks to 6 months, with the majority using a short 2- to 4-week intervention, putting the long-term sustainability of the intervention into question. Three did not have a washout period between the intervention and control groups, and one did not have a run-in phase. The majority of the studies did not assess for changes in body fluid composition, nor did they address patient quality of life. In addition, the available studies are generally of low quality due to unclear randomization, unclear exclusion criteria and the inclusion of low numbers of IDH-prone participants [55].

Only one randomized crossover study (26 HD participants) examined the impact of BVM-guided UF biofeedback on patient symptoms during dialysis. The primary outcome was the percentage reduction in dialysis symptoms, which was 8%. The secondary outcome was a reduction in hypotension [41]. The trial has several weaknesses including short duration (6 weeks), a lack of a washout period, a nonstandard definition of IDH and potential bias due to its industry-funded nature.

Currently, a prospective multi-center triple-arm parallel-group crossover randomized controlled trial is in progress. It is comparing BVM-guided UF biofeedback and blood thermal monitoring temperature biofeedback, to BVM-guided UF and sodium biofeedback, and to standard HD in fluid overloaded HD patients. The primary outcome in this study is the proportion of HD sessions that were complicated with intra- and post-dialytic symptoms related to UF, irrespective of blood pressure. The secondary outcomes include both symptomatic and asymptomatic IDH, using a non-standard definition of a >40 mm Hg drop in systolic blood pressure [59]. In contrast, our study focuses on enrolling symptomatic IDH patients, a standard IDH definition of ≥20 mm Hg, and studying only BVM-guided UF biofeedback without another accompanying biofeedback technology.

We have described our methods for recruitment, randomization, allocation concealment, dialysis intervention, outcome assessment and data collection methods in detail. The study protocol was developed according to the Standard Protocol Items: Recommendations for Interventional Trials (SPIRIT) 2013 [60]. It will be conducted and the results reported following the Consolidated Standards of Reporting Trials (CONSORT) statement [35].

## Trial status

Enrollment began in June 2014 with an expected completion date of July 2015.

## Electronic supplementary material

Additional file 1: **Nursing IDH Management Algorithm.** This is the current local protocol for management of IDH that will be followed by the nurses. (DOCX 102 KB)

## Abbreviations

BNP: brain natriuretic peptide
BVM: blood volume monitoring
CONSORT: Consolidated Standards of Reporting Trials
DRIP: dry-weight reduction in hypertensive hemodialysis patient
ECFV: extracellular fluid volume
HD: hemodialysis
ICFV: intracellular fluid volume
IDH: intradialytic hypotension
SPIRIT: Standard Protocol Items: Recommendations for Interventional Trials
UF: ultrafiltration.
