# Supplementary material for: Ultrafiltration biofeedback guided by blood volume monitoring to reduce intradialytic hypotensive episodes in hemodialysis: study protocol for a randomized controlled trial
Source: Trials. 2014 Dec 10;15:483. doi: 10.1186/1745-6215-15-483 (PMC4295273; doi:10.1186/1745-6215-15-483)
Supplement: Supplementary file 1 — Additional file 1: Nursing IDH Management Algorithm. This is the current local protocol for management of IDH that will be followed by the nurses. (DOCX 102 KB) [file 13063_2014_2376_MOESM1_ESM.docx]

**Additional file 1: Nursing IDH Management Algorithm**

**
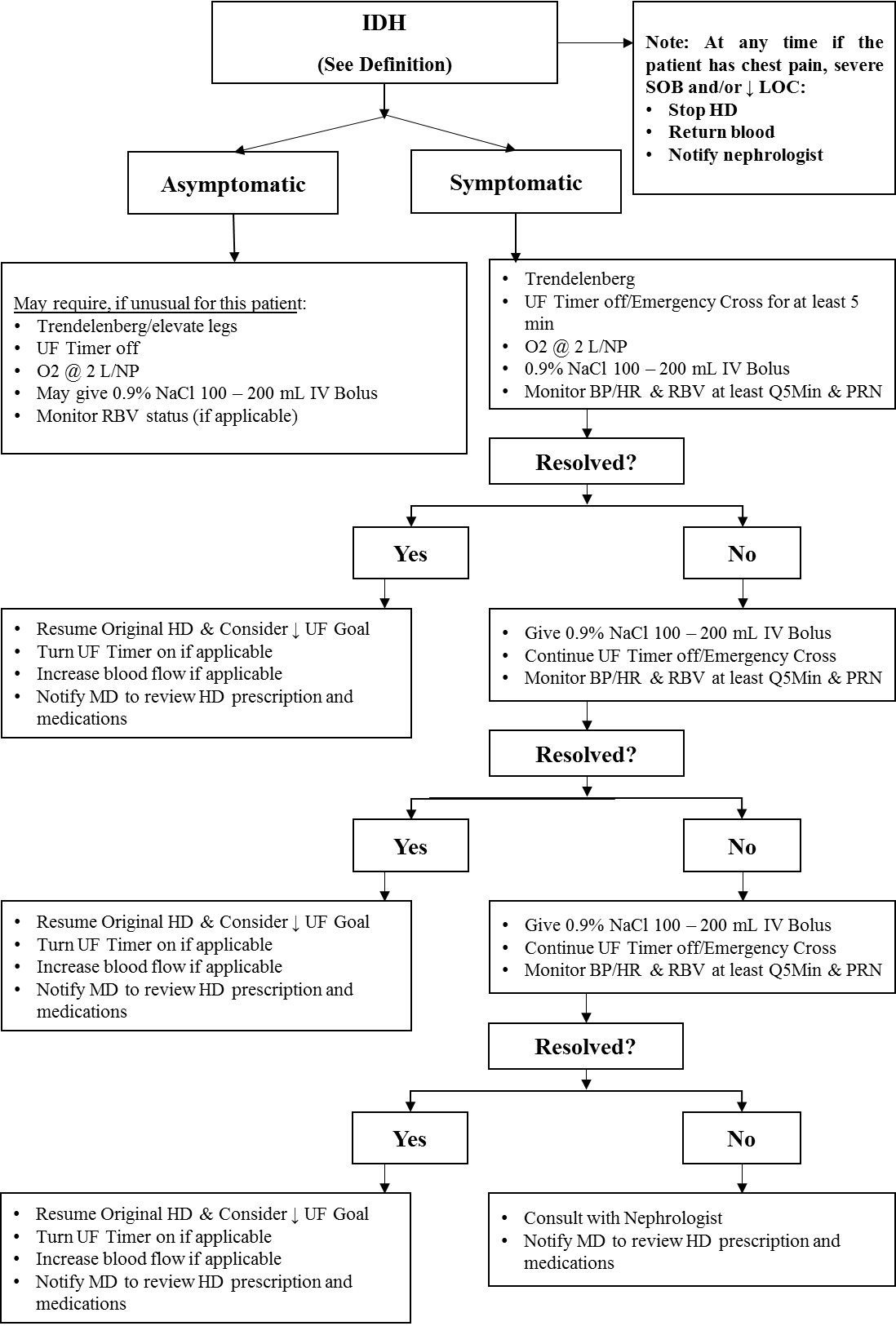
**

**BP = blood pressure; HD = hemodialysis; HR = heart rate; IV = intravenous; LOC = level of consciousness; NP = nasal prongs; PRN = as needed; Q5 = every 5; SOB = short of breath; TW = target weight; UF = ultrafiltration; RBV = relative blood volume.**
